# Hantavirus Expansion Trends in Natural Host Populations in Brazil

**DOI:** 10.3390/v16071154

**Published:** 2024-07-17

**Authors:** José Henrique Fortes Mello, Renata L. Muylaert, Carlos Eduardo Viveiros Grelle

**Affiliations:** 1 Department of Ecology, Institute of Biology, Rio de Janeiro Federal University (UFRJ), Rio de Janeiro 21941-902, Brazil; 2 Knowledge Center for Biodiversity, Belo Horizonte 31270-901, MG, Brazil; 3 Molecular Epidemiology and Public Health Laboratory, School of Veterinary Science—Tāwharau Ora, Massey University, Private Bag 11-222, Palmerston North 4474, New Zealand

**Keywords:** Andes Virus, Araraquara Virus, Juquitiba Virus, *Necromys lasiurus*, *Oligoryzomys nigripes*, cellular automata, SIR model, rodent-borne disease

## Abstract

Hantaviruses are zoonotic agents responsible for causing Hantavirus Cardiopulmonary Syndrome (HCPS) in the Americas, with Brazil ranking first in number of confirmed HCPS cases in South America. In this study, we simulate the monthly spread of highly lethal hantavirus in natural hosts by conjugating a Kermack–McCormick SIR model with a cellular automata model (CA), therefore simultaneously evaluating both in-cell and between-cell infection dynamics in host populations, using recently compiled data on main host species abundances and confirmed deaths by hantavirus infection. For both host species, our models predict an increase in the area of infection, with 22 municipalities where no cases have been confirmed to date expected to have at least one case in the next decade, and a reduction in infection in 11 municipalities. Our findings support existing research and reveal new areas where hantavirus is likely to spread within recognized epicenters. Highlighting spatial-temporal trends and potential expansion, we emphasize the increased risk due to pervasive habitat fragmentation and agricultural expansion. Consistent prevention efforts and One Health actions are crucial, especially in newly identified high-risk municipalities.

## 1. Introduction

Hantaviruses (Order *Bunyavirales*, Family *Hantaviridae*) are negative-stranded RNA zoonotic viruses responsible for two human pathologies: Hemorrhagic Fever with Renal Syndrome (HFRS), associated with Old World hantaviruses found in Europe and Asia; and Hantavirus Cardiopulmonary Syndrome (HCPS), so far restricted to the Americas, associated with New World hantavirus [1,2,3]. In South America, the main HCPS hosts are wild sigmodontine rodents (Family *Cricetidae*) and the main form of spread from hosts to humans is via the inhalation of aerosols loaded with viral particles or by direct contact with blood, saliva, or excreta of infected rodents [2,4]. HCPS is considered an emerging public health issue in Brazil due to its high mortality rates, which vary between 33% and 61%. The first three cases of HCPS in Brazilian territory were reported in 1993, and until 2023, at least 2255 cases of hantavirus infections were confirmed [5,6,7,8,9]. Nine genotypes are currently found in Brazil, of which six are reported to cause HCPS: *Andes orthohantavirus* (ANDV), genotypes *Araraquara virus* (ARQV), *Juquitiba virus* (JUQV), and *Castelo dos Sonhos virus* (CASV); *Rio Mamoré orthohantavirus* (RIOMV); *Anajatuba orthohantavirus* (ANJV); and *Laguna Negra orthohantavirus* (LANV) [6,10,11].

The two main rodent hosts in Brazil are the hairy-tailed bolo mouse *Necromys lasiurus*, mainly found in *Cerrado* areas (a savanna-like formation) and associated with ARQV, and the black-footed pygmy rice rat *Oligoryzomys nigripes*, mostly found in Atlantic Forest areas, and associated with JUQV [12]. However, positive serology has been reported in individuals of more than 16 rodent species in both *Cerrado* and Atlantic Forest biomes [6,12,13,14]. In addition, although most cases have been detected in Brazil, studies have reported that fruit bats and vampire bats (Family *Phyllostomidae*) may be naturally infected, with unknown implications for hantavirus transmission risk to human populations [11,15,16,17]. The habitat generalist behavior of the main rodent hosts [18,19] may explain results from studies that suggest that an increased number of hantavirus infection cases are related with agriculture expansion zones, particularly maize and sugarcane plantations [5,20,21], both because the main host species are expected to respond positively to deforestation and because of the increased number of agricultural workers being exposed to areas where hantavirus is already circulating in rodent communities. There is also evidence of increased HCPS cases during *El Niño* events [22,23], which is probably due to the higher temperatures and dryer climates mostly in *Cerrado* areas, which may facilitate volatilization of aerosols with viral particles.

In this work, we model the spatiotemporal dynamics of hantavirus infection in the two main host species in Brazil in order to assess where we should expect an increase in the number of future cases of hantavirus infection due to spatial expansion in viral circulation in host populations in response to host population abundance and current epidemiological parameters for hantavirus transmission. We simulate the monthly spread of ARQV and JUQV in wild hosts by conjugating a Kermack–McCormick SIR model with a cellular automata model (CA), therefore simultaneously evaluating both in-cell and between-cell infection dynamics in host populations. Several studies suggest that the pervasive deforestation and habitat fragmentation processes in Brazil, due to the expansion of agricultural and cattle areas, lead to an increase in hantavirus rodent host populations [20,24,25]. According to recent estimates of land cover and land use in Brazil [26], as of 2023, approximately 85% of all deforested areas in the country were located either in the Amazon or in the *Cerrado* biomes, mainly driven by agricultural pressure. Assuming this trend is not reversed in the next decade, our central hypothesis is that hantavirus infection should be expected to continue to spread further into *Cerrado* areas, with critical implications for landscape management and public health.

## 2. Materials and Methods

Here, we built our models based on epidemiological parameters for hantavirus transmission in host populations and evaluated the spread of the virus across the country using cellular automata models conjugated with Kermack–McCormick SIR models. We then evaluated the spatial and temporal spread of the virus in host populations, describing epicenters—broader areas where HCPS is more concentrated—and areas of potential expansion and retraction based on our models.

### 2.1. Study Data

We built our model for spatiotemporal spread adapting host population and disease transmission parameters found in the scientific literature. To build our models, we first gathered host abundance data from the ATLANTIC SMALL MAMMALS [27] and the CERRADO SMALL MAMMALS [28] datasets. These databases compile information available in published articles, book chapters, theses, dissertations, monographs, symposium articles, technical reports, and original unpublished data. Prior to our analyses, we ensured all spatial data coordinate reference systems were warped to South America Albers’ Equal Area Conic projection, as it represents areas accurately and is particularly suitable for measuring changes in area size in wide areas such as Brazil (Figure S1). We filtered host population data to include only I) entries with precise geographic coordinates and II) entries from 1990 forward, as the first cases of hantavirus disease in humans in Brazil were reported in 1993. Then, we generated randomized data points to account for pseudoabsences equal to the total number of valid observations, covering all Brazilian territory and with at least 10 km of distance to any other data point, to reduce spatial autocorrelation effects. After removing entries with missing data, we were able to retain 158 valid observations for *Necromys lasiurus* populations and 188 valid observations for *Oligoryzomys nigripes* populations. For each species we transformed abundance/sampling effort to real expected local abundance via quantile regression models [29,30,31], using the median as the model estimate (p_τ0.5_ < 0.05).

Environmental suitability (ES) values were obtained from raster maps developed by Muylaert et al. [20], static at ~1 km of resolution. The ES values used are approximated to the current degree to which the habitat is suitable for the survival, growth, and reproduction of host species based on bioclimatic variables. In order to locally find the effective scale of effect, we defined buffers around each data point with an increasing radius from 500 m to 5000 m, and gathered ES data at each of these scales [32,33]. We then proceeded to evaluate the Spearman correlation between ES values and abundance for each species (Figures S2 and S3). For both *N. lasiurus* and *O. nigripes*, the highest Spearman coefficient was found at the 5000 m radius scale (Spearman’s ρ = 0.573, p < 0.01 and Spearman’s ρ = 0.726, p < 0.01, respectively). Some authors have previously suggested that ES may reveal the areas where the focus species may reach its maximum abundances in the absence of other ecological processes [34,35,36]. However, because the observed relationships between ES and species abundance are seldom linear, we used General Additive Models (GAMs) with Tweedie distribution in order to interpolate abundance related to ES (Figure S4). Therefore, for each species, we generated two raster maps: I) expected abundances predicted by GAMs (Figure S5) and II) binary presence/absence maps based on these expected abundances, using the presence of at least two individuals in a pixel as a threshold for presence. To allow fast computation and balance precision and the generality of our CA models, we set the resolution of our rasters to 10 × 10 km pixels. Quantile regression models and GAMs were built in R [37]; using quantreg [38] and mgcv [39,40] packages, respectively.

We gathered data on confirmed deaths by hantavirus infection from Brazil’s Ministry of Health [41]. We chose to use the number of deaths instead of the total number of cases because, as a consequence of the earlier symptoms of the prodromal phase of hantavirus infections, infected people may often take time to seek health services [42]. Nonetheless, Brazil’s public health policy demands that any suspected death by hantavirus infection must be thoroughly investigated. Therefore, the number of deaths is not only a more accurate metric of hantavirus infection, but it also reflects the access of populations at risk to health services. Thus, it may be interpreted as a proxy for HCPS burden in different parts of Brazil [43,44,45]. Because this dataset only informs the probable municipality where the infection took place, we generated an interpolated raster map of deaths by hantavirus infection using the Inverse Weighted Distance (IDW) algorithm and the number of deaths as an input parameter based on municipality centroids (Figure S6) in QGIS 3.36.0 [46].

In order to binarize this raster map, we used the threshold of 4.5 or above to define the pixels where hantavirus infection is expected to be present. We chose this value because it reaches a good compromise between accuracy and generality when contrasted with observed data in municipality polygons (Figure S7). Finally, we defined all areas where both hantavirus infection and each host species overlapped to set the initial arena for our SIR-CA models to run.

### 2.2. Cellular Automata

Cellular automata (CA) are discrete dynamic computational models based on simple rules capable of simulating physical, biological, or environmental complex phenomena [47,48,49]. In a general form, CA models consist of a two-dimensional lattice of identical cells. Each cell, at any given time *t*, has an associated state determined from a predefined finite set of states. At each time step, each cell is evaluated with a state transition function, commonly based on the state of the cells in its immediate neighborhood. This function may be either deterministic or probabilistic, and the definition of which cells to include in a neighborhood may be stated in several ways. However, the two most important types of neighborhoods are 1) Rook’s case or the von Neumann neighborhood, which includes the cell being evaluated and one or more cells north, south, east and west of that cell; and 2) Queen’s case or the Moore neighborhood, which includes the cell and the eight nearest cells or derivates of it [48,50].

We designed our CA models with two possible states: state 0 (not infected); and state 1 (infected). We defined a deterministic state transition function based on the Moore neighborhood (Queen’s case), where if three or fewer cells in the neighborhood of a focal cell have state 1 at time *t*, then the focal cell state will be updated to state 0 at time *t* + *1*; and updated to state 1 at time *t* + *1* if more than three neighbors are infected.

### 2.3. SIR Models

When modeling the spread of diseases, a widely used starting point is the classic S-I-R model, first proposed by Kermack and McCormick in 1927 [51,52,53]. The general idea of this type of model is that any given population may be subdivided into groups based on the susceptibility to the studied disease. Specifically, in the basic SIR model, *S* denotes the parcel of the population susceptible to the disease, i.e., those not yet exposed to the pathological agent and thus capable of contracting the disease; *I* denotes the number of infected individuals, i.e., the part of population that may spread the disease, and *R*, the number of recovered individuals, i.e., the individuals that are immune to the disease and may no longer spread it further. Considering that the disease does not lead to the death of individuals and that we are dealing with a closed population, the dynamics of these subgroups are commonly evaluated with a set of interactive ordinary differential equations formulated as:

(1) $$\frac{dS}{dt}=-\frac{\beta IS}{N},S\left(0\right)=S_{0}\geq 0,$$

(2) $$\frac{dI}{dt}=\frac{\beta IS}{N}-\gamma I,S\left(0\right)=S_{0}\geq 0,$$

(3) $$\frac{dR}{dt}=\gamma I,R\left(0\right)=R_{0}\geq 0,$$

where *β* is the rate of infection (individuals/time unit) or the transmission rate from infected individuals to susceptible individuals, *γ* is the recovery rate (individuals/time unit), and *S(t)*, *I(t)*, and *R(t)* are the number of individuals of the population at time *t* in each subgroup, so that *S(t)* + *I(t)* + *R(t)* = *N* at any given time, assuming that the host population is stable at equilibrium.

### 2.4. SIR-CA Models

In order to delineate the arena for our SIR-CA models, we defined starting maps for the two main rodent host species by first determining which pixels were within the expected area of occurrence of hantavirus infection cases in Brazil based on the distribution of confirmed deaths by hantavirus [41]. We generated two binary maps based on estimated abundance maps for each host species. For that, we used the threshold of at least two expected individuals in a pixel to define if a host population was present or absent in that given pixel. Finally, we overlayed hantavirus and host population binary maps to generate our modeling arena. Where both hantavirus infection and host presence conditions were found to be co-occurring at *t*_0_, the state of the pixel was defined as 1 (infected), and where neither or only one of these conditions was true, the state of the pixel was defined as 0 (not infected). Whenever a pixel was found to be infected, we ran an SIR model on the estimated rodent host abundance. If the infected local population size (*I*) was smaller than the sum of the susceptible (*S*) and recovered (*R*) individuals at time *t*, then the pixel was considered not infected (state 0). However, because hantavirus spread is directly related to host density, we also defined a rule to account for these density-dependent factors, and thus only pixels where the local abundance value was above half the maximum estimated host abundance (*N. lasiurus*_max/2_ > 8.9828 expected individuals; *O. nigripes*_max/2_ > 9.0618 expected individuals) were valid candidates to be updated prior to applying our CA models.

Each pixel was assigned with a starting population equal to its previously estimated abundance. Assuming that hantavirus infection in wild host populations follows a logistic growth curve, we estimated the average monthly rate of natural host infection *β* as 0.02404, based on the values observed by Figueiredo et al. [54] for *Necromys lasiurus* between 2005 and 2008. This also means that in our model, each time step represents a month. Because of the lack of data regarding recovery rates in natural host populations or indicators of chronic infection, we defined a very low recovery rate value *γ* as 0.0001. With this value for the *β* parameter, it would roughly take a population where 1.2% of the individuals are infected at *t_0_* around 60 years to be completely infected. For example, assume a starting population of 10 *N. lasiurus* individuals, where 75% are at a reproductive stage. Considering that the population presents the vital rates observed by [55,56], it would take 47 generations after the first individual was infected for 99.999% of the population to become infected.

It is reasonable to assume that the infection curves of pixels already infected at *t_0_* should be different from infection curves of pixels only infected at *t_n_*. The rationale behind this premise is that if a given pixel is considered to be infected at *t_0_*, a larger proportion of host population is already expected to be infected, whereas in pixels infected after *t_0_*, the infection should start from zero infected individuals until it reaches an asymptote. Therefore, we designed our SIR models with different initial parameters for each of these scenarios: for pixels infected at *t_0_*, *S* was defined as $\frac{Abundance}{2}-0.1$, *I* as $\frac{Abundance}{2}+0.1$, and *R* as 0. For pixels infected at *t_n_*, initial parameters were defined as S=Abundance−Abundance ∗ 0.02404, I=Abundance ∗ 0.02404, and *R* = 0. Finally, we ran our simulations for each species separately and for both species simultaneously for 120 time steps (months), corresponding to 10 years. SIR models were built in the R environment, using deSolve [57] and reshape2 [58] packages, while CA models were built with custom functions (see Section “Codes” in Supplementary Materials).

## 3. Results

### 3.1. Observed Patterns of Deaths by Hantavirus Infection 1993–2023

**Figure 1:**
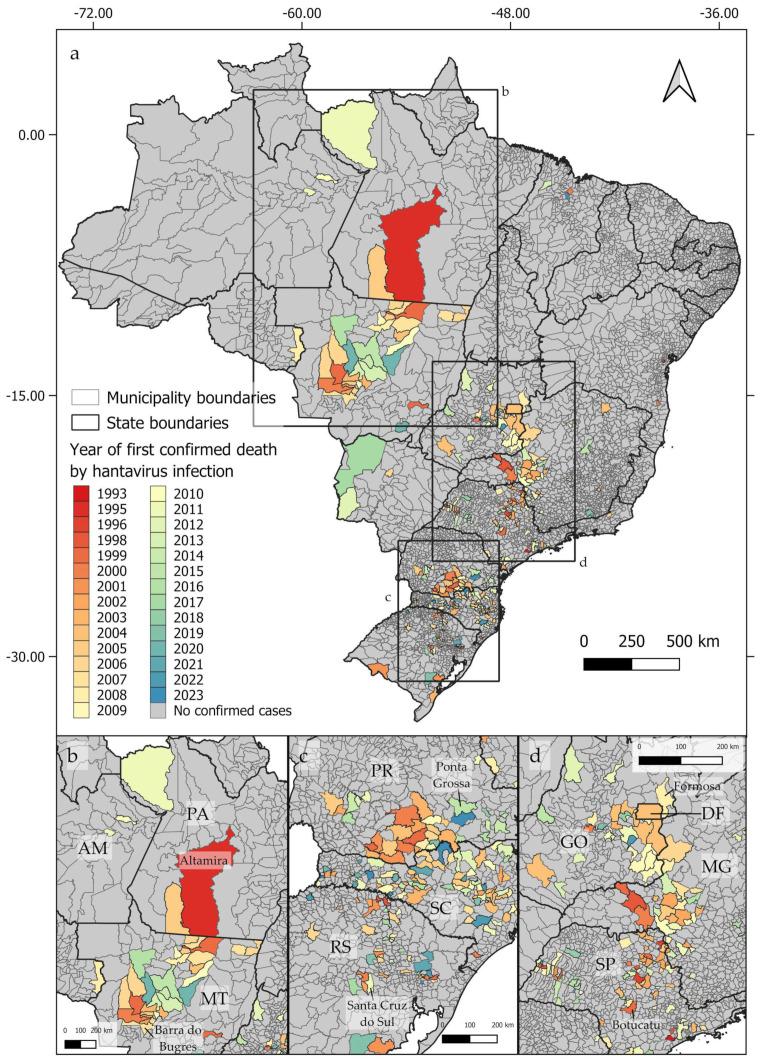
Distribution of confirmed human deaths by hantavirus infection by the year of the first reported cases, showing the three identified epicenters (**a**). The first epicenter (**b**) extends from Altamira, in the State of Pará (PA), to Barra do Bugres, in the State of Mato Grosso (MT). The second epicenter (**c**) extends from Ponta Grossa, located in the State of Paraná (PR), in the north, to Santa Cruz do Sul, located in the State of Rio Grande do Sul (RS), in its southern boundary. The third epicenter (**d**) extends from Formosa, in the State of Goiás (GO), to Botucatu, in the State of São Paulo (SP). State names indicated by two letters: AM = Amazonas; PA = Pará; MT = Mato Grosso; PR = Paraná; SC = Santa Catarina; RS = Rio Grande do Sul; DF = Distrito Federal; GO = Goiás; MG = Minas Gerais; SP = São Paulo.

**Figure 2:**
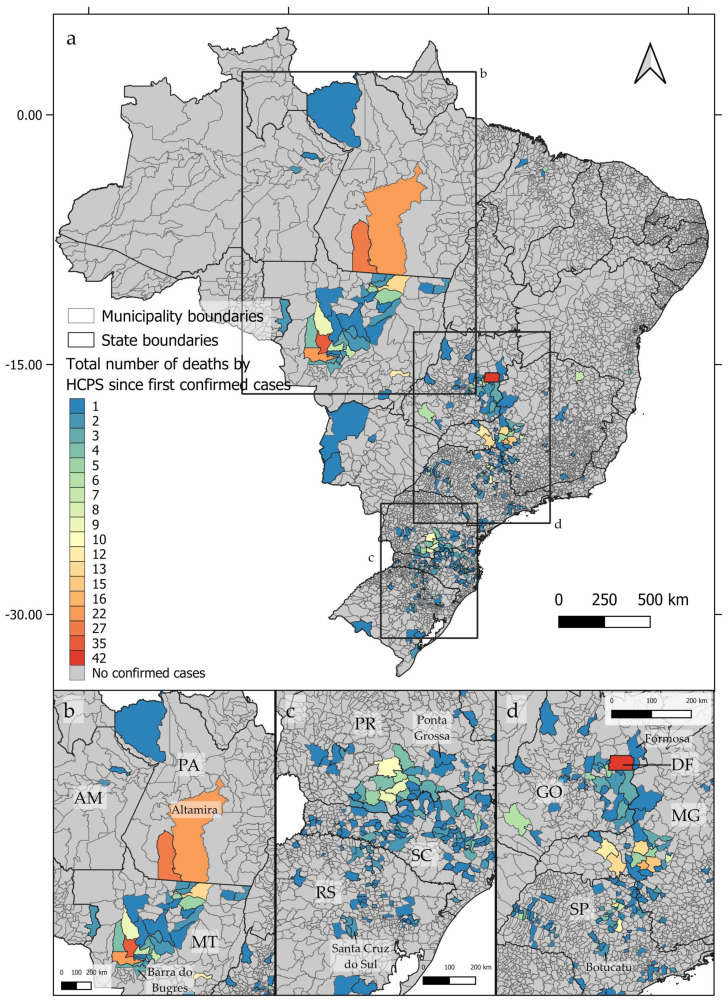
Distribution of confirmed deaths by hantavirus infection by the accumulated number of cases since the first confirmed case at each Brazilian municipality, showing the three identified epicenters (**a**–**d**).

From 1993 to 2023, deaths by hantavirus infection have been confirmed in at least 350 municipalities in Brazil, with three epicenters being identified (Figure 1a). The first epicenter (Figure 1b) occurs in the central-northern regions of Brazil, near the boundaries between the *Cerrado* and Amazon biomes, in the area known as the Amazon Arc of Deforestation. The second nucleus (Figure 1c) may be observed in the southern region of Brazil, mainly across the Paraná and Santa Catarina states, where at least 102 and 117 deaths by HCPS, respectively, were reported to date. The third epicenter extends from the Distrito Federal to the central areas of São Paulo state (Figure 1d). As shown in Figure 2a–d, between 1993 and 2023, the number of deaths by HCPS varied between 1 in most municipalities and 42 in Brasília. We found a positive correlation between the number of years since the first confirmed deaths and the total number of confirmed deaths (r = 0.301, t = 5.887, p < 0.001).

### 3.2. SIR-CA Models

**Figure 3:**
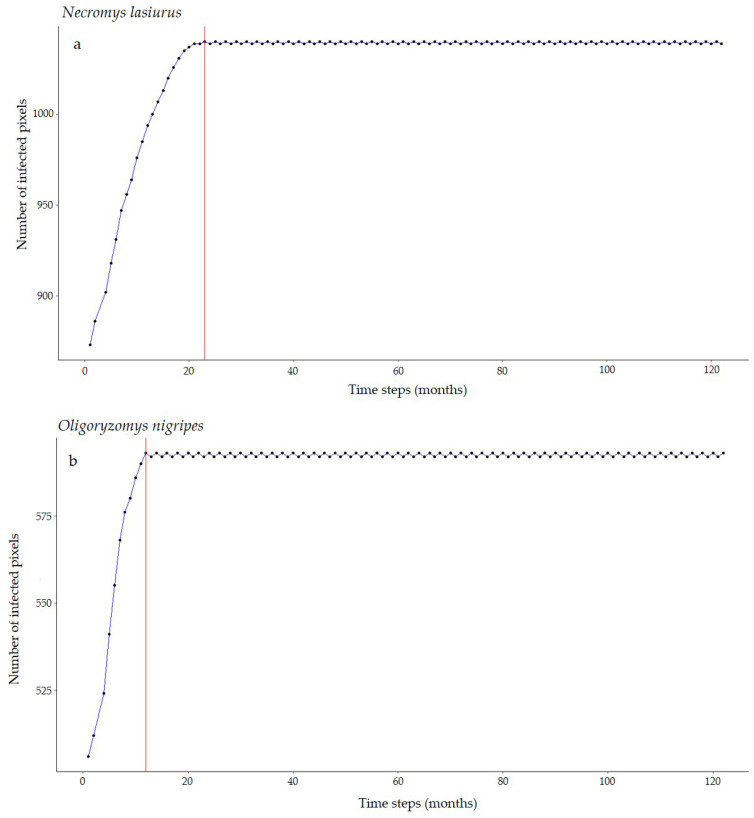
Number of hantavirus-infected pixels over time according to our SIR-CA models for (**a**) *N. lasiurus* and (**b**) *O. nigripes*. Red lines point to the number of time steps (months) it would take to reach this final configuration.

**Figure 4:**
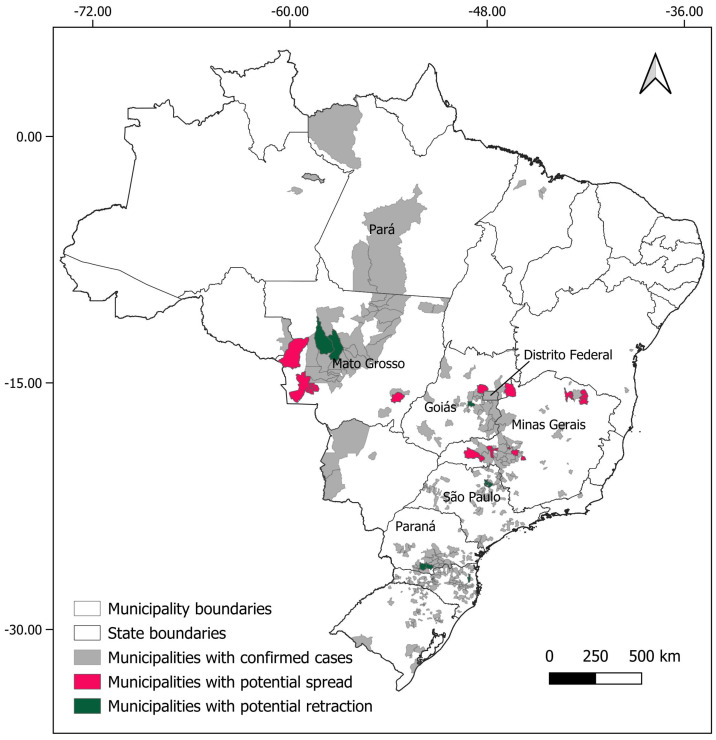
Municipalities with expected spreads (pink) and retractions (green) of hantavirus infection, considering the results for both rodent host species (*Oligoryzomys nigripes* and *Necromys lasiurus*).

For *Necromys lasiurus*, at *t_0_*, we found 873 pixels out of a total of 203,780 pixels in our map, where both hantavirus and *N. lasiurus* were found to be co-occurring. The number of infected pixels at the end of the model run stabilized at 1040 pixels after 23 months (~1.92 years), corresponding to an increased area of 16,700 km² (Figure 3a), or a 19% area expansion. Our models show a continued spread of hantavirus infection, although restricted to *Cerrado* areas (Figure S8a). In particular, invaded areas are adjacent to the surroundings of the Distrito Federal, in the Central-West Region, and the central-western Minas Gerais state. Additional areas of the expected spread of hantavirus infections include one along the international boundaries of Mato Grosso state, and a second, relatively smaller zone in the southeastern areas of Mato Grosso, in the surroundings of the General Carneiro municipality, towards the municipalities of Barra do Garças, Novo São Joaquim, and Pontal do Araguaia (Figure 4). We observed very few areas where hantavirus circulation is expected to disappear, mainly in small regions in Mato Grosso state and São Paulo state (Table S1).

For *Oligoryzomys nigripes*, we found 506 infected pixels at *t_0_*. This number grew until equilibrium at 592 at *t_12_* (1 year, Figure 3b). For this species, our simulations suggest that hantavirus infection is expected to continue to occur and spread in northeastern and eastern areas of Goiás state, in the surroundings of the Distrito Federal. A second area of spread is observed in western Minas Gerais state, where hantavirus infection is expected to occur in municipalities with no previous reported cases, mainly as a consequence of spatial dynamics (Table 1). These municipalities are located in a knowledge gap for the current area of hantavirus distribution (Figure S8b). In São Paulo state, hantavirus infection is expected to continue to be restricted to the north-central areas, while in Paraná state, our model suggests that, although some areas may observe a retraction of hantavirus infection, a westward spread from currently occurring areas should be expected. However, our model also suggests that in Paraná this spread would not include municipalities where no cases have been reported to date (Figure 4, Table S1).

## 4. Discussion

In this study, we developed a mathematical model to evaluate the potential spread of hantavirus reservoirs in Brazil, coupling disease transmission dynamics with the country-wide distribution dynamics of host populations. Although human-to-human spread is possible [59,60], it is considered an extremely rare event, deviating from the main transmission route for hantaviruses, which are through direct contact with or the inhalation of aerosolized rodent excreta. Thus, we focused on modeling the two main hantavirus rodent host species in order to evaluate possible future scenarios in order to better inform decision makers in the more exposed municipalities. We used recently compiled knowledge available on these host species, and a hantavirus infection distribution in Brazil from the first cases through 2023, which increases the robustness of our models and results.

Brazil is a continental-sized country, with most of its natural environments and biomes gravely affected by deforestation and fragmentation due to the continued expansion of mining activity, monoculture farming, and cattle rearing. For instance, just between 2019 and 2023, the *Cerrado* biome lost 33,253.54 km^2^ (an area larger than the entire State of Alagoas), which means a loss of 1.6% of its total area in just five years [26]. In total, the *Cerrado*, which originally covered approximately 22% of Brazilian territory, has already lost 55% of its natural vegetation cover, being currently considered to be one the world’s 25 ecosystems with a high risk of extinction. Similar trends are also observed in the Atlantic Forest, where only 24% of the original vegetation cover is still standing, and in the Amazon Forest, where approximately 20% of the biome area has already been deforested [61,62,63]. Considering the generalist behavior of the two hantavirus rodent hosts evaluated in the present study, there is reason to expect a populational increase in these species in the next decade. Consequently, if these host populations indeed become infected, there would also be an increase in the exposure of humans to viral particles. Although our models are not spatially explicit in a strict sense, not including effect interactions, environmental heterogeneity, or land cover dynamics, our results provide a suitable step forward in building more applicable models for hantaviruses by integrating spatial and temporal processes to explain infection dynamics in natural populations. For instance, we found a positive correlation between years since the first confirmed deaths and the total number of confirmed cases, which suggests that these are areas where hantavirus is a chronically present in the environment. This is further corroborated by comparing the maps of years since the first confirmed cases and the number of cases since the first confirmed deaths, which clearly shows at least three discernible compartments. The first epicenter extends between the states of Pará, in the North Region, and Mato Grosso, in the Central-West Region. We detected an area of potential spread in the southern regions of Mato Grosso state, in the vicinity of General Carneiro, a municipality ranked in the top 20 predicted risk areas by Muylaert et al. [20]. We have reason to believe that two variants may be occurring in this epicenter, CASV and ARQV: the area of distribution of the main host for ARQV, *Necromys lasiurus*, does not extend into the Amazon. However, the municipalities of Altamira and Novo Progresso, both in Pará state, have two of the largest numbers of total confirmed cases (35 and 27, respectively). In the risk rank framework developed by [20], Novo Progresso figures in the top 10 high-risk municipalities for hantavirus infection risk in Brazil, while Altamira was ranked 23rd. In this region, some authors have suggested that a highly probable candidate as a host for CASV is the pygmy rice rat *Oligoryzomys utiaritensis* [64,65]. Unfortunately, there is a nearly complete lack of data on this species to date, and thus, we were unable to include it in our models.

A second epicenter extends from Brasília, in the Federal District, to central areas of São Paulo state, in the Southeast Region of Brazil. At least two variants may also be found to be co-occurring in this epicenter, since the areas of distribution of *O. nigripes*, the main host for JUQV, and *N. lasiurus* overlap in this region. The third epicenter is observed in the South Region of Brazil, extending from the state of Paraná to the state of the Rio Grande do Sul. In this case, we believe that hantavirus infection may be mainly related to ARQV, since, of the two main hosts, only *O. nigripes* occurs in the region. The South Region is where most deaths by HCPS were consistently reported for 2021, 2022, and 2023. In 2023, 9 out of a total of 18 HCPS deaths were reported, but data for the period between 2023 and 2024 is still under investigation [66].

The results of our models for *N. lasiurus* suggest that a slight increase in hantavirus infection risk should be expected in the next 10 years, with at least 16 municipalities where no cases have been reported to date under suspicion, in the states of Mato Grosso, Goiás, Minas Gerais, and São Paulo (Table 1). Our model also suggests that there is a possibility that ARQV could spread to the Santa Cruz department in central eastern Bolívia. For *O. nigripes*, our model suggests that, similarly to the trends observed for *N. lasiurus*, a slight increase in the distribution of hantavirus infection should be expected to occur in the next decade. At least 18 municipalities, in the states of Mato Grosso, São Paulo, Minas Gerais and Santa Catarina, where deaths by hantavirus have never been previously reported could be exposed to this increase in spread (Table S1). Considering both species, our models suggest that at least 22 municipalities where no cases have been previously reported should expect to experience at least one case of hantavirus infection in the next 10 years. On the other hand, we also identified at least 11 municipalities with previously reported cases where infection is expected to subside. The region around the Distrito Federal, central-western and northeastern Minas Gerais state are of particular interest for public health action to take place, as in these areas both ARQV and JUQV rodent hosts co-occur, which greatly increases risks of human exposure to at least one variant. Importantly, these are the same areas that previous studies point out as being of high risk [5,20,67].

It is important to note that in its current implementation, our model does not account for potential effects of climatic change or projections of future deforestation, both mechanisms known to affect the rodent host species. However, we believe our model is flexible enough to incorporate these processes. For instance, our starting maps are built over abundance maps generated over environmental suitability maps. Therefore, it would be possible to assess ES changes in different climatic change scenarios for host species and evaluate the expected abundance, which, in turn, would be used to feed our models. Hantavirus infection also shows signs of seasonal fluctuations, mostly related to crop harvesting periods. During harvest season, rural worker population increases, while rodent host populations are attracted to storage grains, thus increasing human exposure to viral particles present in the environment [5,21]. Including temporal variation in host populations may further increase the accuracy of our models and we intend to implement and evaluate this in the near future. However, we believe that even in its current form, our model could be used to inform better health policies.

One particular shortfall identified was the lack of data regarding *Oligoryzomys utiaritensis* ES or abundance, which prevented us from applying our model to the Amazon. We have reason to suspect that the number of cases in Altamira and Novo Progresso, both in Pará state, will continue to be high, and it is not unreasonable to consider that a new outbreak may occur in the next few years. This particular municipality is within the so-called Amazon Arc of Deforestation, a large area of a massive expansion of monoculture and cattle farming, in the same region where previous studies have found naturally hantavirus-infected phyllostomid bats [15]. Moreover, the health systems in these municipalities are not well prepared to deal with large outbreaks, and, for instance, were among those that experienced systemic collapse during the peak of the COVID-19 pandemic in 2021. The same could happen in the event of co-occurring outbreaks of more than one disease, especially considering the long hospitalization period in intensive care units that treatment for HCPS commonly demands. Thus, our model could also be further improved by including factors such as human population density or projected changes in land usage as conditional parameters or thresholds.

In recent years, Brazilian legislators have passed several bills that reduce environmental protection [68,69], and consequently increased the risks of future zoonotic outbreaks. To deal with the increased risks of new zoonotic diseases, the country must reverse the political agenda for the environment of the last decade. Because of the substantial evidence linking hantavirus disease and landscape change [70], it is essential that the One Health approach becomes integrated and operationalized by stakeholders and decision makers [71,72].

In conclusion, our findings corroborate previous works and shows novel areas of potential hantavirus spread, suggesting that within all three identified epicenters, hantavirus should be expected to consistently circulate and expand more often than subside. Moreover, we expect these trends to be further amplified by continued habitat fragmentation and agricultural expansion [70], which means hantavirus prevention must be prioritized, especially in settings where the populations at risk could be inadvertently exposed such as in the detected novel areas of expansion highlighted in this study. Fortunately, at least for zoonotic diseases with higher potential to negatively affect humans such as Dengue fever, yellow fever, and hantavirus disease, Brazil has monitoring and handling protocols in place [73]. After a suspected case is confirmed, state agencies implement local active surveillance programs in the region and its surroundings. Notably, most states with chronic hantavirus infection that implemented annual training programs for health workers reported decreases in fatal cases. Hantavirus disease cases are relatively rare but frequently fatal. Thus, these monitoring programs must be continued and strengthened to prevent potential outbreaks and losses of human lives.

## Figures and Tables

**Table 1:** Hantavirus spread trends in Brazilian municipalities. Pathogenic hantavirus dynamics over 10 years according to SIR-CA models. Municipalities of consistent spread are shown in Table S1.

Epicenter	State	Municipality	Pathogenic Hantavirus Dynamics
Central	Minas Gerais	Rio Paranaiba	Expansion
Central	Minas Gerais	Iraí de Minas	Expansion
Central	Minas Gerais	Cruzeiro da Fortaleza	Expansion
Central	Mato Grosso	Pontes e Lacerda	Expansion
Central	Mato Grosso	Araputanga	Expansion
Central	Minas Gerais	Buritis	Expansion
Central	Mato Grosso	Comodoro	Expansion
Central	Minas Gerais	Estrela do Indaiá	Expansion
Central	Mato Grosso	Figueirópolis d’Oeste	Expansion
Central	Minas Gerais	Indaiabira	Expansion
Central	Minas Gerais	Indianópolis	Expansion
Central	Mato Grosso	Indiavaí	Expansion
Central	Mato Grosso	Jauru	Expansion
Central	Minas Gerais	Nova Ponte	Expansion
Central	Goiás	Padre Bernardo	Expansion
Central	Minas Gerais	Porteirinha	Expansion
Central	Minas Gerais	Prata	Expansion
Central	Minas Gerais	Salinas	Expansion
Central	Minas Gerais	Serrania	Expansion
Central	Minas Gerais	Taiobeiras	Expansion
Central	Mato Grosso	Tesouro	Expansion
Central	Minas Gerais	Veríssimo	Expansion
Central	São Paulo	Sertãozinho	Reduction
Central	São Paulo	Ribeirão Preto	Reduction
Central	São Paulo	Pontal	Reduction
Central	Goiás	Anápolis	Reduction
Central	São Paulo	Barrinha	Reduction
Central	Mato Grosso	Brasnorte	Reduction
Central	Mato Grosso	Nova Maringá	Reduction
South	Paraná	Bituruna	Reduction
South	Santa Catarina	Arroio Trinta	Reduction
South	Santa Catarina	Blumenau	Reduction
South	Paraná	Coronel Domingos Soares	Reduction

## Data Availability

The original data and codes used to generate our results are available from the author’s GitHub repository at https://github.com/JosMello/Zoonotic_modeling (accessed on 19 June 2024).

## Supplementary Materials

The following supporting information can be downloaded at: https://www.mdpi.com/article/10.3390/v16071154/s1, Figure S1: Map of Brazil depicting the country’s biomes, states, and all municipalities where at least one case was confirmed up to 2023 (gray areas). All 26 Brazilian states and the Federal District are shown on the map. In the North Region: AC = Acre; AM = Amazonas; RO = Rondônia; RR = Roraima; AP = Amapá; PA = Pará; TO = Tocantins. In the Northeastern Region: MA = Maranhão; PI = Piauí; CE = Ceará; RN = Rio Grande do Norte; PB = Paraíba; PE = Pernambuco; AL = Alagoas; SE = Sergipe; BA = Bahia. In the Central-Western Region: MT = Mato Grosso; MS = Mato Grosso do Sul; GO = Goiás; DF = Distrito Federal. In the Southeastern Region: SP = São Paulo; MG = Minas Gerais; ES = Espírito Santo; RJ = Rio de Janeiro. In the South Region: PR = Paraná; SC = Santa Catarina; RS = Rio Grande do Sul; Figure S2: Spearman correlations between environmental suitability and abundance for *Necromys lasiurus* at different buffer scales. Highest Spearman τ found at a 5000 m radius; Figure S3: Spearman correlations between environmental suitability and abundance for *Oligoryzomys nigripes* at different buffer scales. Highest Spearman τ found at a 5000 m radius; Figure S4: GAM plots between predicted abundance and environmental suitability for the main hantavirus hosts in Brazil, in areas where local populations were empirically sampled. (**a**) *Necromys lasiurus*, (**b**) *Oligoryzomys nigripes.* Each point represents an observation, the gray shaded areas are the GAMs smooths confidence intervals, and the small bars at the horizontal axis are the values of each observation; Figure S5: Expected abundance and distribution maps. (**a**) *Necromys lasiurus* expected abundance varied between 0 and 17,96 individuals, (**b**) *Oligoryzomys nigripes* expected abundance varied between 0 and 9,69 individuals. As a consequence of a lack of distributional and abundance data, we were unable to run our models for *O. utiaritensis*, the main host for Castelo dos Sonhos virus (CASV); Figure S6: Distribution map of confirmed deaths by hantavirus infection. Values interpolated via the IDW algorithm, based on the centroid of the municipalities; Figure S7: Binary map based on the interpolated number of confirmed deaths, using a threshold value of 4.5; Figure S8: Final configuration of the hantavirus spread map according to the SIR-CA model. Over 10 years, for *Necromys lasiurus* (**a**), 272 pixels, covering an area of 16,700 km^2^), became infected while 105 pixels, covering an area of 10,500 km^2^, became uninfected, resulting in an overall expected increase of 6200 km^2^. For *Oligoryzomys nigripes* (**b**), 125 pixels, covering an area of 12,500 km^2^, became infected, while 39 pixels, covering 3900 km^2^, became uninfected, resulting in an overall expected increase of 8600 km^2^. Light pink pixels are the areas where both hantavirus infection and host species are currently present, red pixels are the areas to where infection is expected to spread, black pixel are areas where hantavirus infection is expected to recede; Table S1: Model projections on hantavirus infection. (+) represents municipalities with no previous confirmed cases where infection is expected to spread, (-) represents municipalities with previous confirmed cases where infection is expected to subside, and (N) are municipalities with previous confirmed cases where infection is expected to be present. Municipality risk values and risk rank are from Muylaert et al. [20]; Codes: Functions used to evaluate SIR values and SIR-CA models.
