# Supplementary material for: Hantavirus Expansion Trends in Natural Host Populations in Brazil
Source: Viruses. 2024 Jul 17;16(7):1154. doi: 10.3390/v16071154 (PMC11281686; doi:10.3390/v16071154)
Supplement: Supplementary file 1 [file viruses-16-01154-s001.zip › viruses-3103793-supplementary.pdf]

viruses

Supplementary Materials:

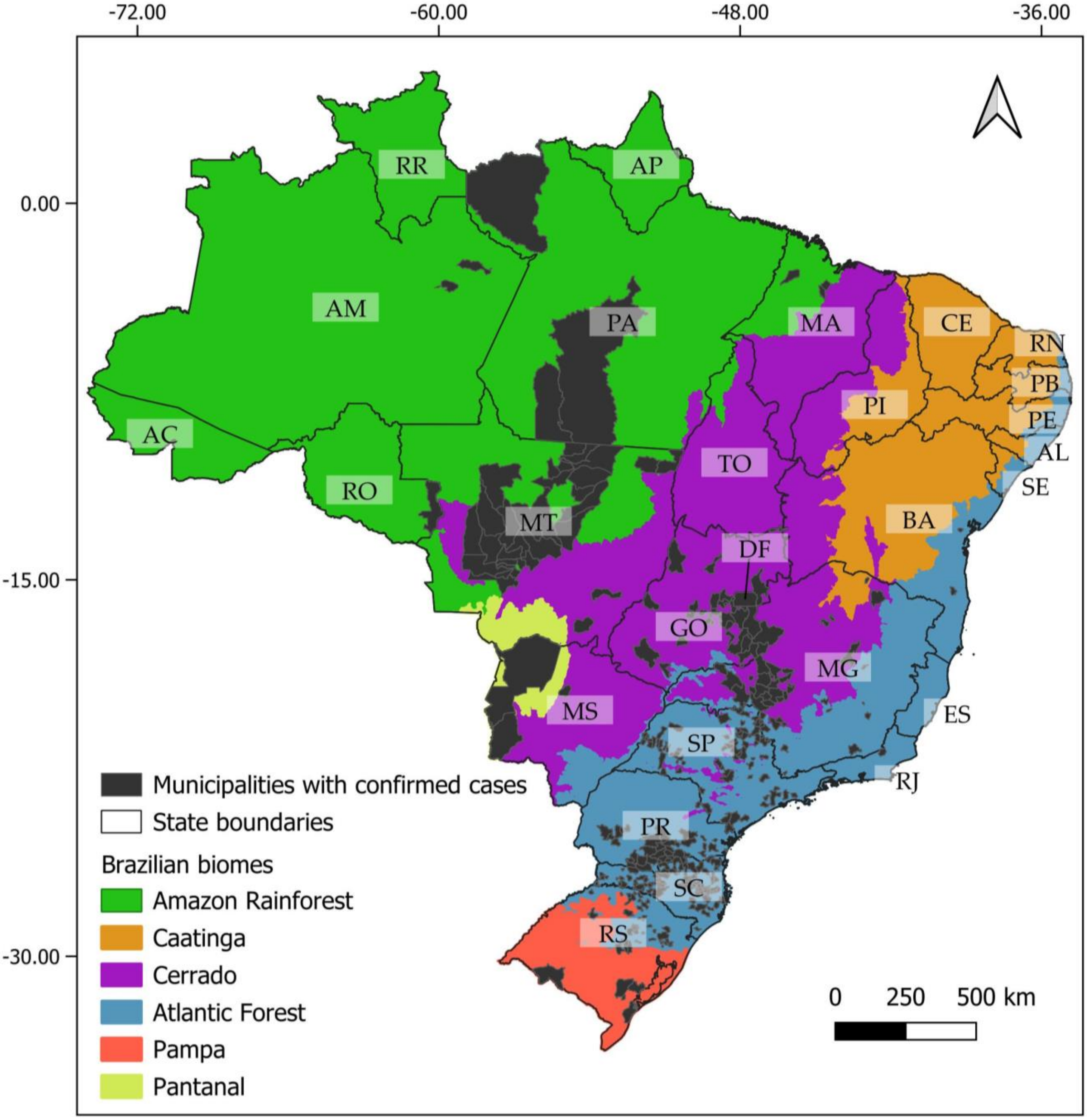

Figure S1: Map of Brazil depicting the country's biomes, states, and all municipalities where at least one case was confirmed until 2023 (gray areas). All 26 Brazilian states and the Federal District are shown in the map. In the North Region: AC = Acre; AM = Amazonas; RO = Rondônia; RR = Roraima; AP = Amapá; PA = Pará; TO = Tocantins. In the Northeastern Region: MA = Maranhão; PI = Piauí; CE = Ceará; RN = Rio Grande do Norte; PB = Paraíba; PE = Pernambuco; AL = Alagoas; SE = Sergipe; BA = Bahia. In the Central-Western Region: MT = Mato Grosso; MS = Mato Grosso do Sul; GO = Goiás; DF = Distrito Federal. In the Southeastern Region: SP = São Paulo; MG = Minas Gerais; ES = Espírito Santo; RJ = Rio de Janeiro. In the South Region: PR = Paraná; SC = Santa Catarina; RS = Rio Grande do Sul.

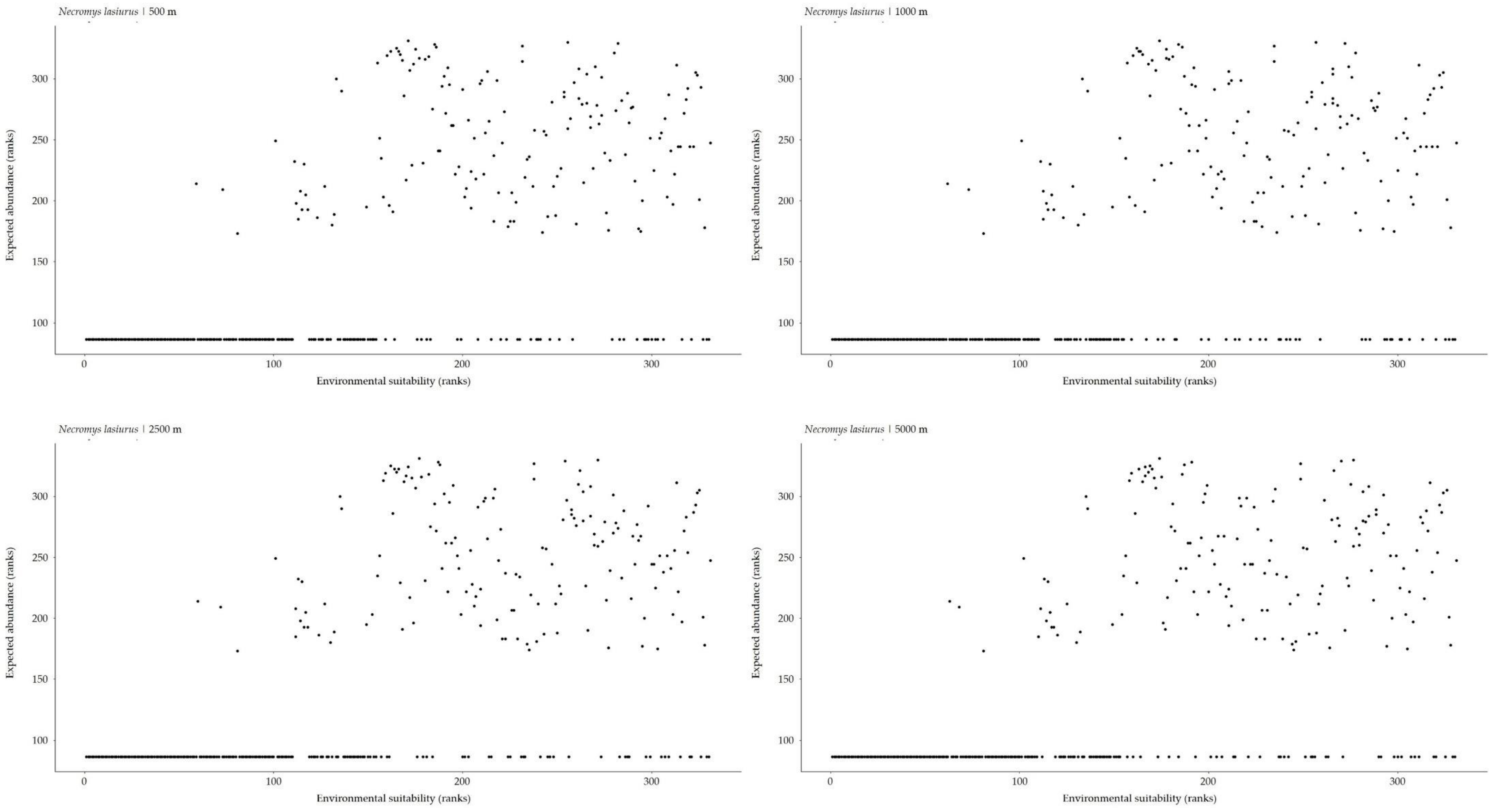

Figure S2: Spearman correlations between environmental suitability and abundance for *Necromys lasiurus* at different buffer scales. The highest Spearman τ was found at a 5000 m radius.

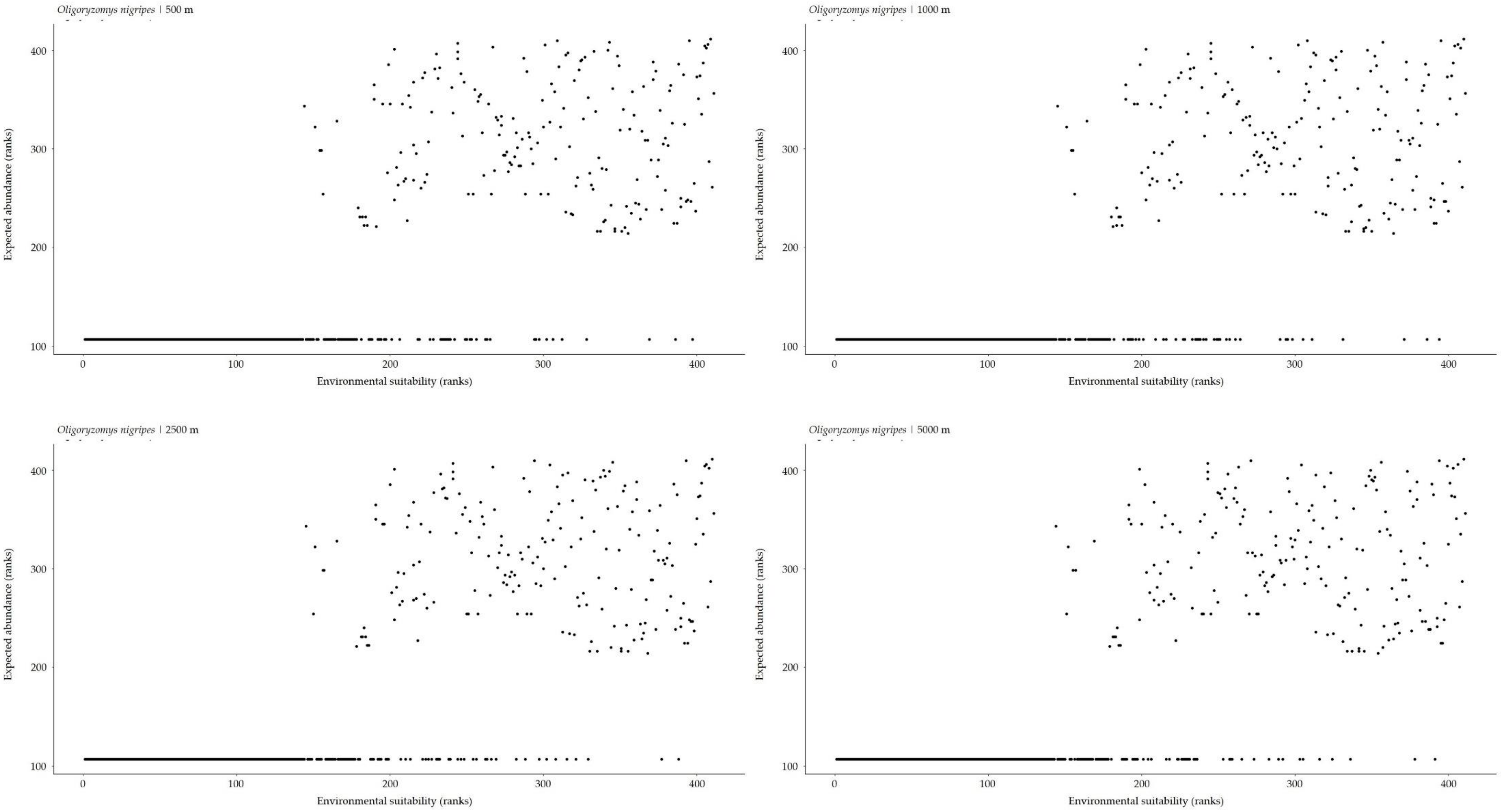

Figure S3: Spearman correlations between environmental suitability and abundance for *Oligoryzomys nigripes* at different buffer scales. The highest Spearman τ was found at a 5000 m radius.

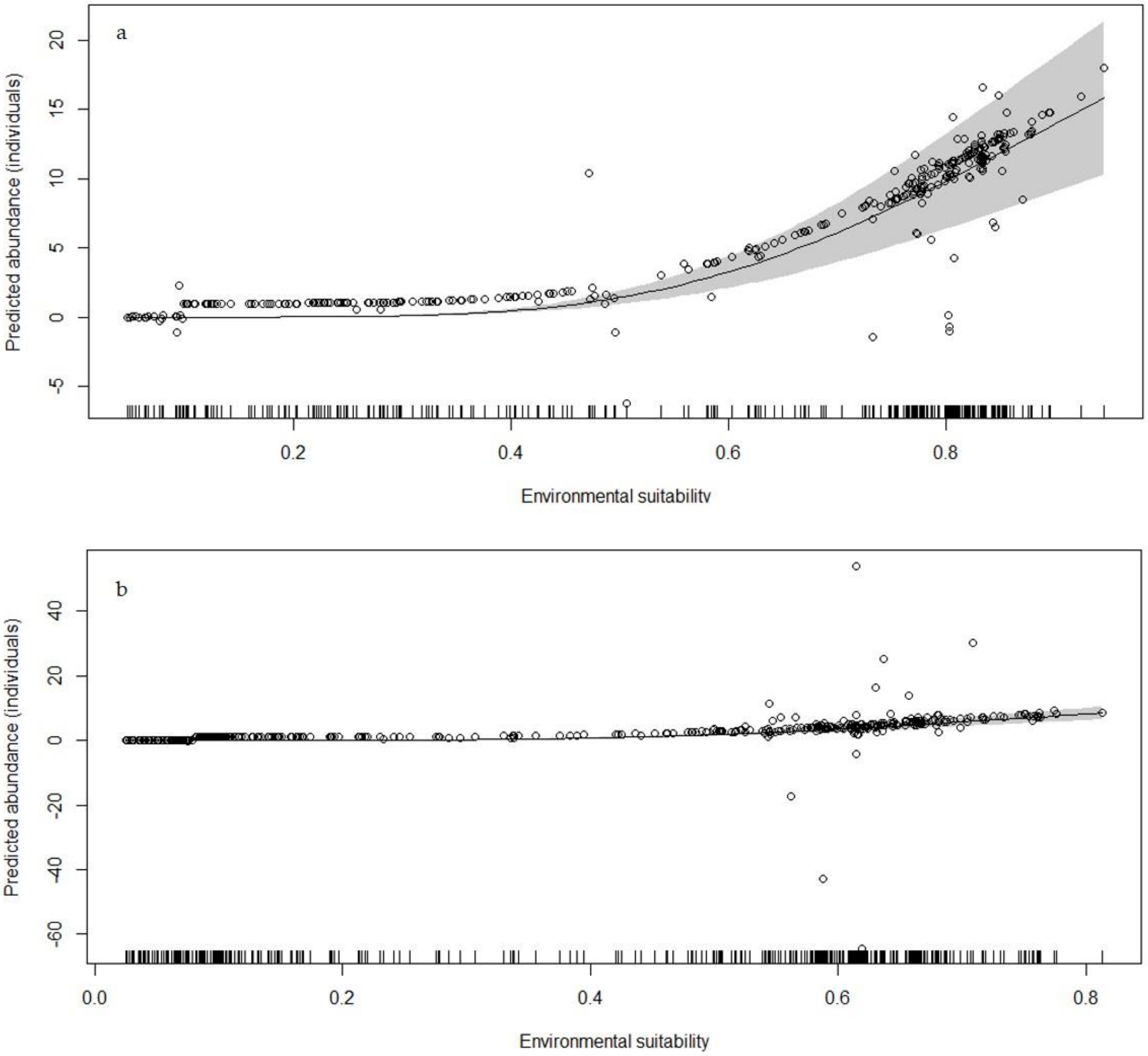

Figure S4: GAM plots between predicted abundance and environmental suitability for the main hantavirus hosts in Brazil, in areas where local populations were empirically sampled. **(a)** *Necromys lasiurus*, **(b)** *Oligoryzomys nigripes*. Each point represents an observation, the gray shaded areas are the GAMs smooths confidence intervals, and the small bars at the horizontal axis are the values of each observation.

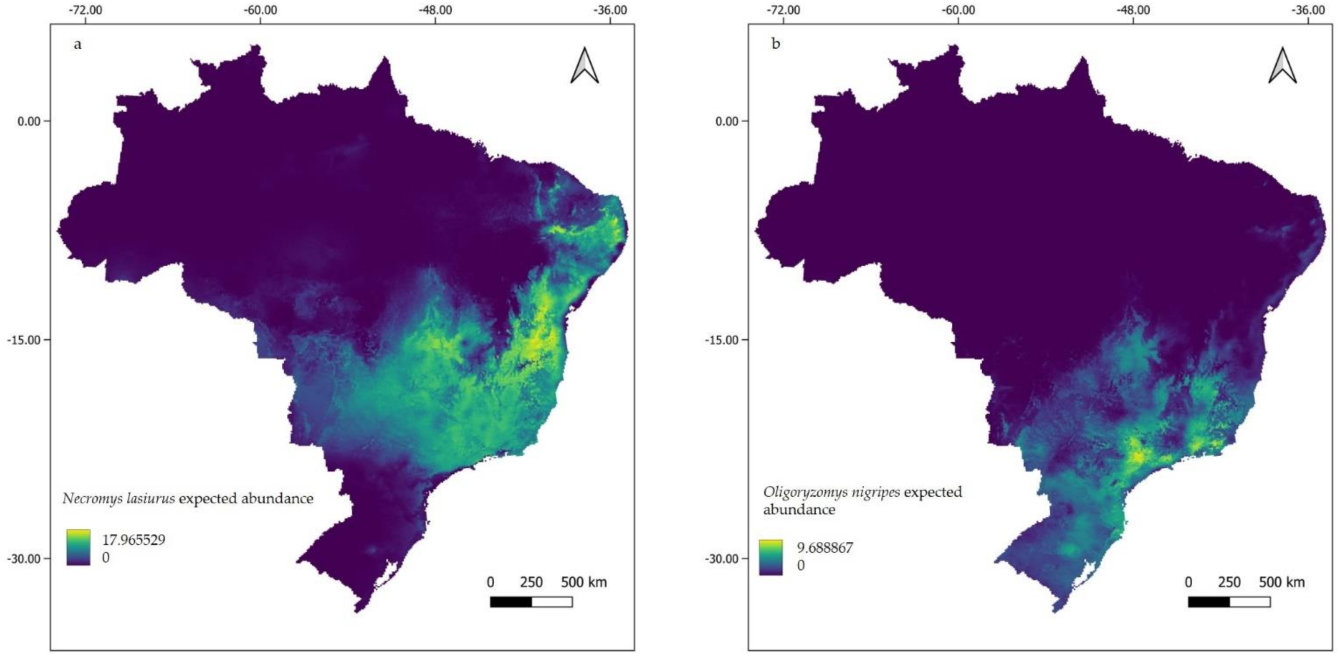

Figure S5: Expected abundance and distribution maps. (**a**) *Necromys lasiurus* expected abundance varied between 0 and 17,96 individuals, (**b**) *Oligoryzomys nigripes* expected abundance varied between 0 and 9,69 individuals. As a consequence of a lack of distributional and abundance data, we were unable to run our models for *O. utiaritensis*, the main host for Castelo dos Sonhos virus (CASV).

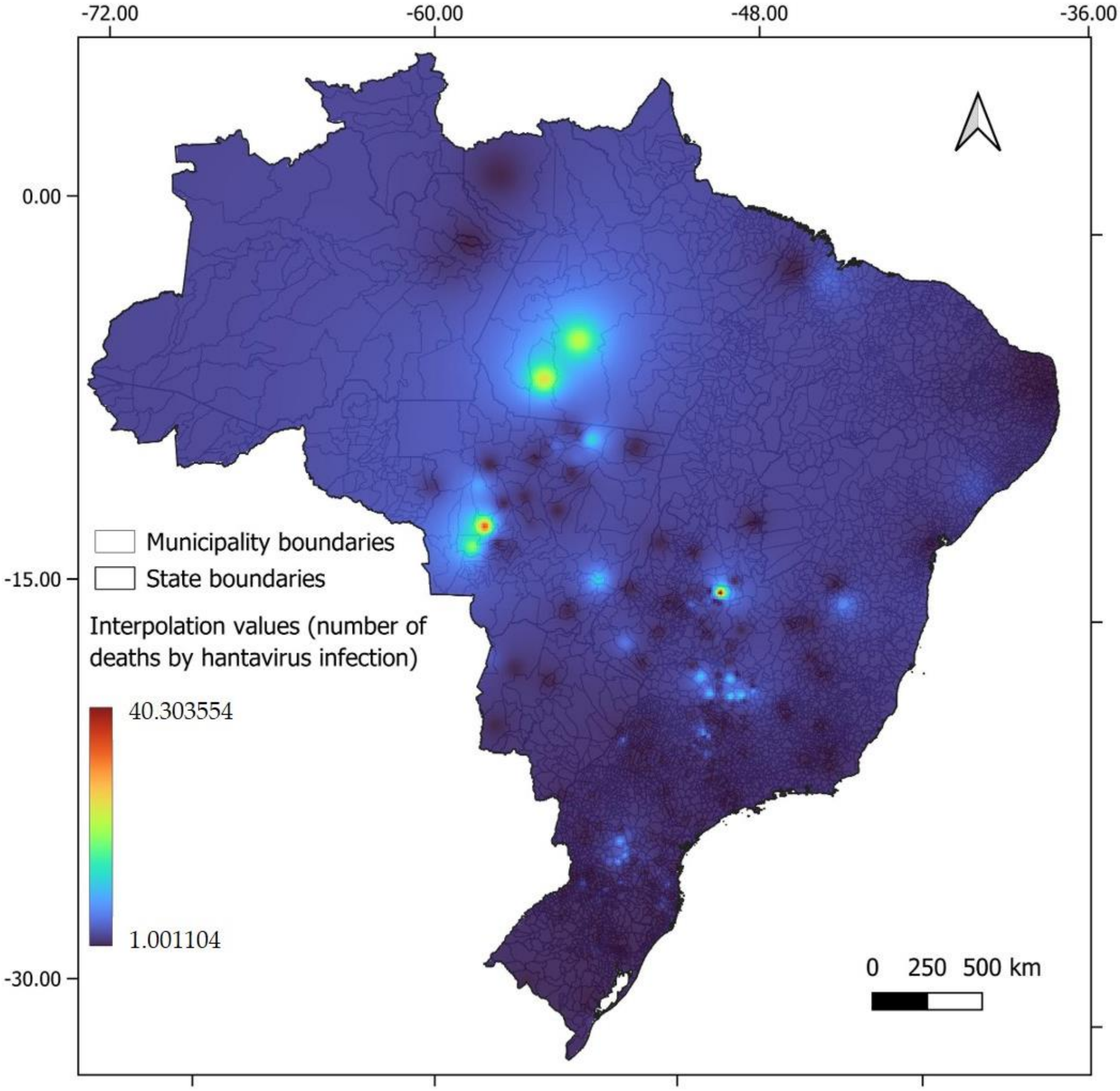

Figure S6: Distribution map of confirmed deaths by hantavirus infection. Values interpolated via the IDW algorithm, based on the centroid of the municipalities.

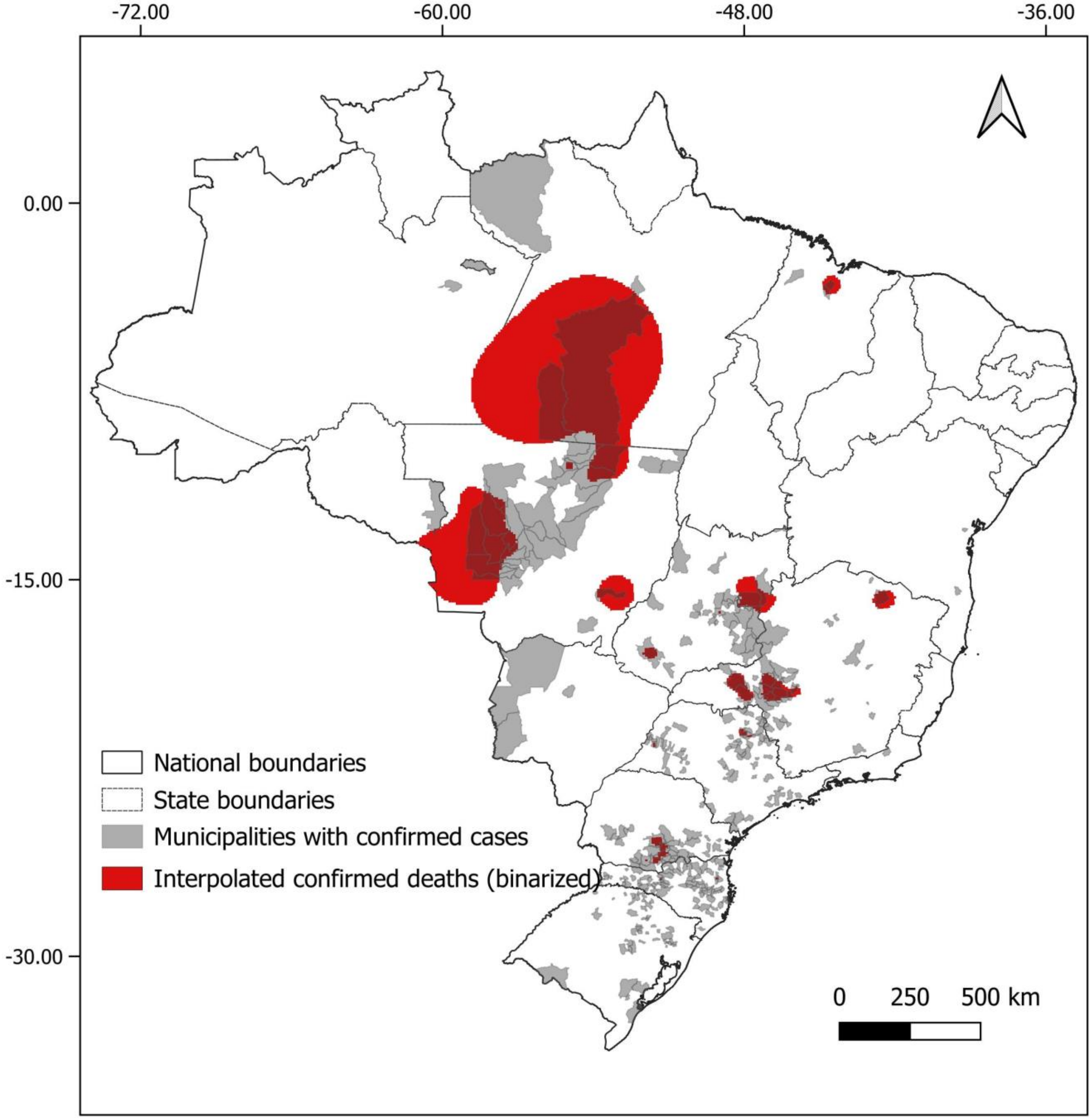

Figure S7: Binary map based on the interpolated number of confirmed deaths, using a threshold value of 4.5.

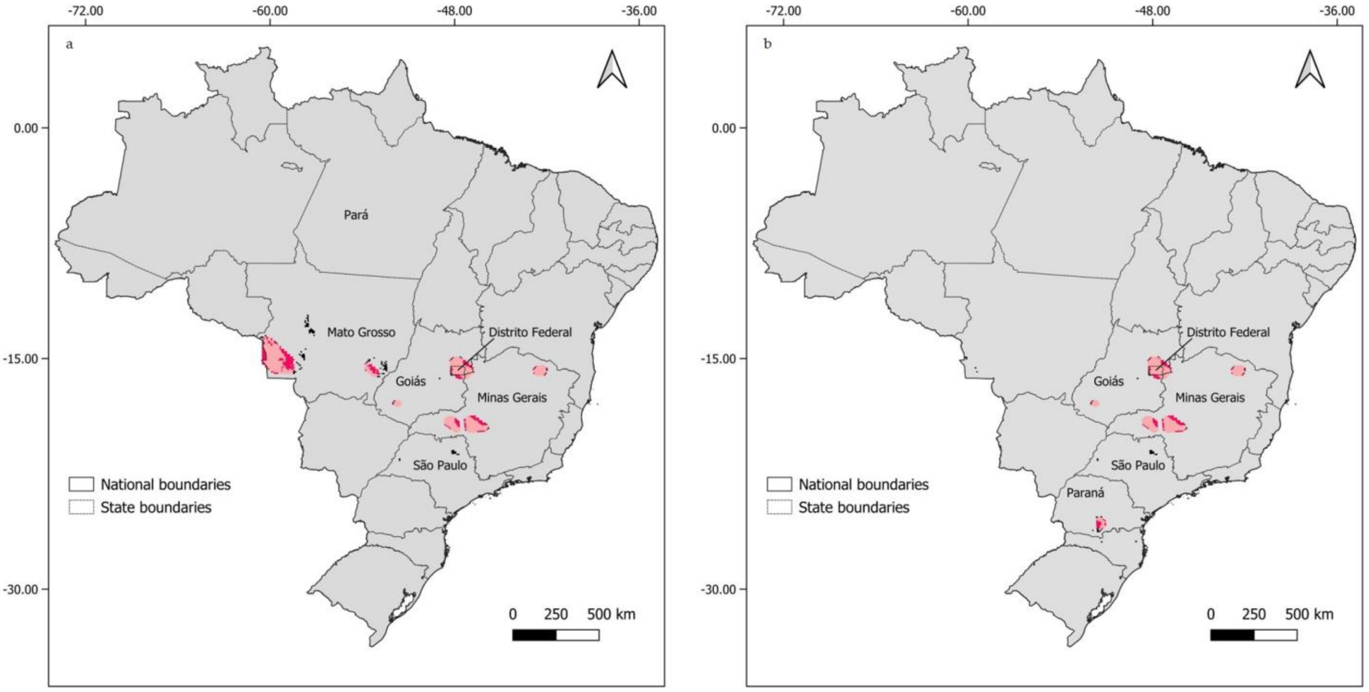

Figure S8: Final configuration of the hantavirus spread map according to the SIR-CA model. Over 10 years, for *Necromys lasiurus* **(a),** 272 pixels, covering an area of 16,700 km², became infected while 105 pixels, covering an area of 10,500 km², became uninfected, resulting in an overall expected increase of 6,200 km². For *Oligoryzomys nigripes* **(b)**, 125 pixels, covering an area of 12,500 km², became infected, while 39 pixels, covering 3,900 km², became uninfected, resulting in an overall expected increase of 8,600 km². Light pink pixels are the areas where both hantavirus infection and host species are currently present, red pixels are the areas to where infection is expected to spread, black pixel are areas where hantavirus infection is expected to recede.

Table S1: Model projections on hantavirus infection. (+) represents municipalities with no previous confirmed cases where infection is expected to spread, (-) represents municipalities with previous confirmed cases where infection is expected to subside, and (N) are municipalities with previous confirmed cases where infection is expected to be present. Municipality risk values and risk ranks are from Muylaert et al. [20].

State	Municipality	Hantavirus infection	Risk*	Risk rank*
Distrito Federal	Brasília	N	0.771	1
Mato Grosso	Campo Novo do Parecis	N	0.749	2
Minas Gerais	Patrocínio	N	0.727	3
Minas Gerais	Araxá	N	0.671	4
Mato Grosso	Tangará da Serra	N	0.643	5
Paraná	Cruz Machado	N	0.616	6
São Paulo	Sertaozinho	-	0.602	7
Minas Gerais	São Gotardo	N	0.579	8
Pará	Novo Progresso	N	0.555	9
Minas Gerais	Ibiá	N	0.521	10
São Paulo	Ribeirao Preto	-	0.506	11
Santa Catarina	Concórdia	N	0.493	12
Mato Grosso	Nova Olímpia	N	0.481	13
Mato Grosso	Diamantino	N	0.479	14
Minas Gerais	Uberlândia	N	0.441	15

Paraná	Rio Azul	N	0.439	16
Minas Gerais	Uberaba	N	0.439	16
São Paulo	Pontal	-	0.436	18
Mato Grosso	General Carneiro	N	0.426	19
Paraná	Bituruna	-	0.381	20
Minas Gerais	São Tomás de Aquino	N	0.351	21
Goiás	Campo Alegre de Goiás	N	0.333	22
Pará	Altamira	N	0.330	23
Minas Gerais	Perdizes	N	0.324	24
Paraná	Mallet	N	0.312	25
Santa Catarina	Ipumirim	N	0.311	26
Paraná	União da Vitória	N	0.309	27
Mato Grosso	Peixoto de Azevedo	N	0.305	28
Paraná	Inácio Martins	N	0.304	29
Santa Catarina	Arroio Trinta	-	0.293	30
São Paulo	São Carlos	N	0.293	30
Goiás	Anápolis	-	0.289	32
Mato Grosso	Terra Nova do Norte	N	0.287	33
Santa Catarina	Blumenau	-	0.285	34
Minas Gerais	Rio Paranaiba	+	0.276	35
Santa Catarina	Videira	N	0.265	36
Minas Gerais	Campos Altos	N	0.255	37
Paraná	Irati	N	0.254	38
Mato Grosso	Santo Afonso	N	0.253	39
Paraná	Mangueirinha	N	0.251	40
Mato Grosso	Sapezal	N	0.233	42
Mato Grosso	Barra dos Bugre	N	0.232	43
Santa Catarina	Joaçaba	N	0.229	44
Paraná	Pinhão	N	0.229	44
Santa Catarina	Alfredo Wagner	N	0.227	46
Paraná	Guarapuava	N	0.220	47
São Paulo	Araraquara	N	0.218	48
Paraná	Porto Vitória	N	0.213	49
Goiás	Valparaíso de Goiás	N	0.212	50
Minas Gerais	Patos de Minas	N	0.205	51
São Paulo	Ibaté	N	0.203	52
Mato Grosso	Marcelândia	N	0.201	54
São Paulo	Itirapuã	N	0.199	55
Mato Grosso	São José do Rio Claro	N	0.199	55
Paraná	Palmas	N	0.197	57
Paraná	Coronel Domingos Soares	-	0.196	58
Santa Catarina	São Bonifácio	N	0.195	59
Santa Catarina	Ituporanga	N	0.193	60
Goiás	Jataí	N	0.193	60
Minas Gerais	Tapira	N	0.193	60
Santa Catarina	Peritiba	N	0.191	63

Santa Catarina	Arabutã	N	0.190	64
São Paulo	Barrinha	-	0.189	65
Santa Catarina	Caçador	N	0.188	67
Santa Catarina	Luzerna	N	0.188	67
Santa Catarina	Leoberto Leal	N	0.179	69
Santa Catarina	Taió	N	0.178	70
Minas Gerais	São João Batista do Glória	N	0.177	71
Minas Gerais	Passos	N	0.174	72
Santa Catarina	Jaborá	N	0.170	73
Santa Catarina	Vitor Meireles	N	0.169	74
Mato Grosso	Brasnorte	-	0.168	75
São Paulo	Orlandia	N	0.168	75
Paraná	Prudentópolis	N	0.167	78
Paraná	São Mateus do Sul	N	0.163	81
Mato Grosso	Colíder	N	0.162	82
Goiás	Cristalina	N	0.162	82
Rio Grande do Sul	Santa Cruz do Sul	N	0.159	84
São Paulo	Mogi das Cruzes	N	0.158	86
Santa Catarina	Rio do Campo	N	0.158	86
Santa Catarina	Santo Amaro da Imperatriz	N	0.158	86
Goiás	Luziânia	N	0.157	89
São Paulo	São Paulo	N	0.156	90
São Paulo	Cássia dos Coqueiros	N	0.154	91
Santa Catarina	Porto União	N	0.152	92
São Paulo	Dumont	N	0.150	94
Santa Catarina	Pouso Redondo	N	0.149	95
Santa Catarina	Ibicaré	N	0.145	96
Santa Catarina	Itaiópolis	N	0.145	96
Santa Catarina	Iomerê	N	0.142	99
Goiás	Santo Antônio do Descoberto	N	0.141	100
Minas Gerais	Serra do Salitre	N	0.141	100
Santa Catarina	Vidal Ramos	N	0.140	102
Mato Grosso	Nortelândia	N	0.136	104
Mato Grosso	Matupá	N	0.135	105
Mato Grosso	Denise	N	0.133	107
Minas Gerais	São Sebastião do Paraíso	N	0.133	107
Santa Catarina	Rio dos Cedros	N	0.130	109
Santa Catarina	Major Gercino	N	0.128	111
Rio Grande do Sul	Caxias do Sul	N	0.126	113
Santa Catarina	Papanduva	N	0.126	113
Minas Gerais	Guimarania	N	0.125	115
Minas Gerais	Santa Juliana	N	0.125	115
Santa Catarina	Abelardo Luz	N	0.124	119
Goiás	Cidade Ocidental	N	0.124	119
Santa Catarina	Chapecó	N	0.122	122
Santa Catarina	Rio Negrinho	N	0.119	124

Minas Gerais	Sacramento	N	0.119	124
Santa Catarina	Ponte Serrada	N	0.117	128
Santa Catarina	Aurora	N	0.115	130
Mato Grosso	Nova Marilândia	N	0.115	130
Mato Grosso	Guarantã do Norte	N	0.114	133
Minas Gerais	Coromandel	N	0.111	135
Minas Gerais	Iraí de Minas	+	0.111	135
São Paulo	Mirandópolis	N	0.109	138
Santa Catarina	Mirim doce	N	0.108	140
Goiás	Goiania	N	0.107	141
Mato Grosso	Santa Terezinha	N	0.107	141
São Paulo	Guariba	N	0.104	144
São Paulo	Santa Rosa de Viterbo	N	0.104	144
Santa Catarina	Presidente Nereu	N	0.103	146
Santa Catarina	Iraceminha	N	0.102	148
Goiás	Catalão	N	0.101	151
Santa Catarina	Rio do Sul	N	0.099	156
Minas Gerais	Paracatu	N	0.097	158
Paraná	Quatro Barras	N	0.097	158
Santa Catarina	Salete	N	0.095	163
Santa Catarina	Canoinhas	N	0.091	167
Rio Grande do Sul	Carlos Barbosa	N	0.091	167
Minas Gerais	Pratinha	N	0.091	167
Paraná	Paulo Frontin	N	0.089	172
Santa Catarina	Rio Fortuna	N	0.089	172
Paraná	Clevelândia	N	0.087	174
Santa Catarina	Macieira	N	0.087	174
Santa Catarina	Descanso	N	0.086	177
São Paulo	Serrana	N	0.086	177
Rio de Janeiro	Cantagalo	N	0.085	179
Minas Gerais	Cruzeiro da Fortaleza	+	0.084	181
Rio Grande do Sul	São Domingos do Sul	N	0.084	181
São Paulo	Lucélia	N	0.083	184
Mato Grosso	Nova Maringá	-	0.082	186
São Paulo	Patrocínio Paulista	N	0.081	188
Paraná	Catanduvas	N	0.079	191
Mato Grosso	Nova Mutum	N	0.079	191
Goiás	Alexânia	N	0.078	196
Minas Gerais	Ibiraci	N	0.078	196
São Paulo	Matão	N	0.078	196
Santa Catarina	Palhoça	N	0.078	196
Goiás	Senador Canedo	N	0.078	196
Santa Catarina	Urubici	N	0.077	205
Paraná	Paula Freitas	N	0.076	207
Santa Catarina	Lacerdópolis	N	0.075	210
Rondônia	Vilhena	N	0.075	210

Santa Catarina	Calmon	N	0.074	212
Goiás	Corumbá de Goiás	N	0.074	212
Goiás	Ipameri	N	0.074	212
São Paulo	Dracena	N	0.073	216
Santa Catarina	Rio do Oeste	N	0.072	220
São Paulo	Jardinópolis	N	0.070	227
São Paulo	Mococa	N	0.069	232
Rio Grande do Sul	Cacique doble	N	0.068	235
Santa Catarina	Ibiam	N	0.068	235
Mato Grosso	Lucas do Rio Verde	N	0.068	235
Paraná	Honório Serpa	N	0.067	242
Santa Catarina	Campo Alegre	N	0.066	243
Santa Catarina	Presidente Getúlio	N	0.066	243
São Paulo	Cajuru	N	0.065	245
Minas Gerais	Delta	N	0.065	245
Mato Grosso	Pontes e Lacerda	+	0.065	245
Santa Catarina	Atalanta	N	0.064	248
Mato Grosso	União do Sul	N	0.064	248
Paraná	São José dos Pinhais	N	0.063	252
São Paulo	Eldorado	N	0.062	258
Goiás	Cocalzinho de Goiás	N	0.061	261
São Paulo	Luís Antônio	N	0.061	261
São Paulo	Mariápolis	N	0.061	261
São Paulo	Paulicéia	N	0.061	261
São Paulo	Santa Ernestina	N	0.061	261
Minas Gerais	Carmo do Paranaiba	N	0.060	267
Santa Catarina	São João Batista	N	0.060	267
Santa Catarina	Agronômica	N	0.059	273
Rio Grande do Sul	Canguçu	N	0.059	273
Paraná	Cascavel	N	0.059	273
Santa Catarina	Gaspar	N	0.059	273
São Paulo	Miguelópolis	N	0.059	273
Rio Grande do Sul	Passo Fundo	N	0.059	273
Rio Grande do Sul	São Lourenço do Sul	N	0.059	273
Minas Gerais	Arceburgo	N	0.058	284
Minas Gerais	Guarda-Mor	N	0.058	284
Minas Gerais	São José da Barra	N	0.058	284
Goiás	Águas Lindas de Goiás	N	0.057	287
Santa Catarina	Joinvile	N	0.057	287
São Paulo	Nova Guataporanga	N	0.057	287
Mato Grosso	Feliz Natal	N	0.056	292
São Paulo	Junqueirópolis	N	0.056	292
Goiás	Silvânia	N	0.056	292
Paraná	Antonio Olinto	N	0.055	297
Santa Catarina	Bocaina do Sul	N	0.055	297
Rio Grande do Sul	Gramado	N	0.055	297

São Paulo	Pacaembu	N	0.055	297
Rio Grande do Sul	Picada Café	N	0.055	297
Rio Grande do Sul	Viadutos	N	0.055	297
Goiás	Pirenópolis	N	0.054	306
Minas Gerais	Romaria	N	0.054	306
São Paulo	Altinópolis	N	0.053	311
Santa Catarina	Campos Novos	N	0.053	311
Santa Catarina	Orleaes	N	0.053	311
São Paulo	Ribeirão Bonito	N	0.053	311
Santa Catarina	Major Vieira	N	0.052	316
Rio Grande do Sul	Erechim	N	0.051	320
Mato Grosso	Itaúba	N	0.050	323
Goiás	Acreúna	N		
São Paulo	Aguai	N		
Maranhão	Anajatuba	N		
Goiás	Anicuns	N		
Mato Grosso	Araputanga	+		
Maranhão	Arari	N		
Paraná	Araucária	N		
São Paulo	Arco-íris	N		
Rio Grande do Sul	Arvorezinha	N		
São Paulo	Assis	N		
Rio Grande do Sul	Barao de Cotegipe	N		
São Paulo	Barra do Turvo	N		
São Paulo	Barretos	N		
São Paulo	Bastos	N		
São Paulo	Borá	N		
São Paulo	Botucatu	N		
Minas Gerais	Buritis	+		
Goiás	Cachoeira Alta	N		
Bahia	Camacan	N		
Minas Gerais	Campestre	N		
São Paulo	Campinas	N		
Paraná	Campo Magro	N		
Goiás	Campos Belos	N		
Rio Grande do Sul	Candelária	N		
Santa Catarina	Capinzal	N		
Amazonas	Careiro da Várzea	N		
Rio Grande do Sul	Centenário	N		
Mato Grosso	Comodoro	+		
Mato Grosso	Confresa	N		
Minas Gerais	Coroaci	N		
Santa Catarina	Coronel Freitas	N		
Santa Catarina	Coronel Martins	N		
Mato Grosso do Sul	Corumbá	N		
São Paulo	Cotia	N		

Rio Grande do Sul	David Canabarro	N
Paraná	Diamante do Sul	N
Minas Gerais	Diamantina	N
Rio Grande do Sul	Dois Irmãos	N
Rio Grande do Sul	Entre Rios do Sul	N
Minas Gerais	Estrela do Indaiá	+
São Paulo	Fartura	N
Paraná	Fernandes Pinheiro	N
Mato Grosso	Figueirópolis d'Oeste	+
São Paulo	Flórida Paulista	N
Rio Grande do Sul	Fontoura Xavier	N
Goiás	Formosa	N
São Paulo	Franca	N
Santa Catarina	Guaramirim	N
Paraná	Guaraniaçu	N
Santa Catarina	Guatambú	N
São Paulo	Iepê	N
Rio Grande do Sul	Igrejinha	N
Rio Grande do Sul	Ijuí	N
Rio Grande do Sul	Ilópolis	N
Minas Gerais	Indaiabira	+
Minas Gerais	Indianópolis	+
Mato Grosso	Indiavaí	+
Goiás	Inhumas	N
São Paulo	Iporanga	N
Goiás	Itaberaí	N
Santa Catarina	Itapiranga	N
Rio Grande do Sul	Itapuca	N
São Paulo	Itararé	N
Minas Gerais	Itatiaiuçu	N
São Paulo	Ituverava	N
Mato Grosso	Jauru	+
Mato Grosso	Juara	N
Minas Gerais	Juiz de Fora	N
São Paulo	Juquitiba	N
Rio Grande do Sul	Lajedão	N
Paraná	Laranjal	N
Santa Catarina	Lontras	N
São Paulo	Macatuba	N
São Paulo	Mairiporã	N
Minas Gerais	Mamonas	N
São Paulo	Martinópolis	N
Minas Gerais	Materlândia	N
Paraná	Matinhos	N
Minas Gerais	Matutina	N
São Paulo	Monte Alto	N

Goiás	Montes Claros de Goiás	N
Rio Grande do Sul	Morro Redondo	N
São Paulo	Motuca	N
Rio Grande do Norte	Natal	N
Goiás	Nerópolis	N
Goiás	Nova Crixás	N
Santa Catarina	Nova Erechim	N
Maranhão	Nova Olinda do Maranhão	N
Rio Grande do Sul	Nova Petrópolis	N
Minas Gerais	Nova Ponte	+
Rio Grande do Sul	Nova Roma do Sul	N
Santa Catarina	Nova Trento	N
Mato Grosso	Nova Ubiratã	N
Pará	Oriximiná	N
São Paulo	Oscar Bressane	N
São Paulo	Osvaldo Cruz	N
Goiás	Padre Bernardo	+
Paraná	Palmeira	N
São Paulo	Panorama	N
Minas Gerais	Pará de Minas	N
São Paulo	Pederneiras	N
Mato Grosso	Pedra Preta	N
Santa Catarina	Petrolândia	N
Santa Catarina	Pinheiro Preto	N
Goiás	Pires do Rio	N
Paraná	Ponta Grossa	N
Santa Catarina	Ponte Alta do Norte	N
Minas Gerais	Ponte Nova	N
Minas Gerais	Porteirinha	+
Mato Grosso do Sul	Porto Murtinho	N
Minas Gerais	Prata	+
Rio Grande do Sul	Putinga	N
Paraná	Quedas do Iguaçu	N
São Paulo	Quitana	N
Paraná	Quitandinha	N
São Paulo	Registro	N
São Paulo	Rio Claro	N
São Paulo	Rio das Pedras	N
Rio Grande do Sul	Rio Grande	N
Mato Grosso do Sul	Rio Negro	N
São Paulo	Salesópolis	N
Minas Gerais	Salinas	+
Rio Grande do Sul	Sananduva	N
São Paulo	Santa Cruz da Conceição	N
Santa Catarina	Santa Helena	N

Rio Grande do Sul	Santa Maria do Herval	N
São Paulo	Santa Mercedes	N
Rio Grande do Sul	Santana do Livramento	N
São Paulo	Santo Antônio da Alegria	N
Bahia	Santo Antônio de Jesus	N
Rio Grande do Sul	Santo Antônio do Palma	N
São Paulo	São João do Pau d'Alho	N
Paraná	São José da Boa Vista	N
São Paulo	São Manuel	N
Santa Catarina	São Miguel do Oeste	N
Minas Gerais	Serrania	+
Rio Grande do Sul	Sinimbu	N
São Paulo	Tabatinga	N
Minas Gerais	Taiobeiras	+
Santa Catarina	Tangará	N
Rio Grande do Sul	Tapejara	N
Mato Grosso	Tapurah	N
Mato Grosso	Tesouro	+
Santa Catarina	Timbó Grande	N
Rio Grande do Sul	Três Arroios	N
Rio Grande do Sul	Três Coroas	N
Rio Grande do Sul	Três Passos	N
São Paulo	Tupã	N
Minas Gerais	Tupaciguara	N
São Paulo	Tupi Paulista	N
Santa Catarina	Turvo	N
Goiás	Uruaçu	N
Santa Catarina	Urupema	N
Rio Grande do Sul	Vanini	N
Santa Catarina	Vargem Bonita	N
Rio Grande do Sul	Vera Cruz	N
Minas Gerais	Veríssimo	+
Rio Grande do Sul	Vespasiano Correa	N
Goiás	Vianópolis	N
Santa Catarina	Xanxerê	N

*Values are from Muylaert et al. [20].

Codes: Functions used to evaluate SIR values and SIR-CA models

```
sir_equations <- function(time, variables, parameters) {
with(as.list(c(variables, parameters)),
                                  {
dS <- -beta * I * S
dI <-  beta * I * S - gamma * I
dR <-  gamma * I
return(list(c(dS, dI, dR)))
})}

num_neighbors <- function(grid, i, j){
sum(grid[max(1, i-1):min(nrow(grid), i+1),
max(1, j-1):min(ncol(grid), j+1)]) - grid[i, j]}

update_grid <- function(grid){
                      new_grid <- grid
                      for (i in 1:nrow(grid)) {
                            for (j in 1:ncol(grid)) {
neighbors <- num_neighbors(grid, i, j)
new_grid[i, j] <- ifelse(grid[i, j] == 1,
ifelse(neighbors <= 3, 0, 1),
ifelse(neighbors > 3, 1, 0))
}
     }
                      new_grid}
```
